# Chemoresistance to Concanamycin A1 in Human Oral Squamous Cell Carcinoma Is Attenuated by an HDAC Inhibitor Partly via Suppression of Bcl-2 Expression

**DOI:** 10.1371/journal.pone.0080998

**Published:** 2013-11-20

**Authors:** Tamotsu Kiyoshima, Hisato Yoshida, Hiroko Wada, Kengo Nagata, Hiroaki Fujiwara, Makiko Kihara, Kana Hasegawa, Hirotaka Someya, Hidetaka Sakai

**Affiliations:** 1 Laboratory of Oral Pathology, Division of Maxillofacial Diagnostic and Surgical sciences, Faculty of Dental Science, Kyushu University, Fukuoka, Japan; 2 Department of Orthodontics, Division of Oral Health, Growth and Development, Faculty of Dental Science, Kyushu University, Fukuoka, Japan; 3 Department of Endodontology and Operative Dentistry, Division of Oral Rehabilitation, Faculty of Dental Science, Kyushu University, Fukuoka, Japan; 4 Department of Removable Prosthodontics, Division of Oral Rehabilitation, Faculty of Dental Science, Kyushu University, Fukuoka, Japan; Louisiana State University Health Sciences center, United States of America

## Abstract

V-ATPase is involved in the acidification of the microenvironment around/in solid tumors, such as oral squamous cell carcinoma (OSCC). V-ATPase is thought to induce tumor invasion and multi-drug resistance in several malignant tumors, and it also contributes to maintaining the intracellular pH under an acidic microenvironment by inducing proton extrusion into the extracellular medium. However, there is little information regarding the effects of V-ATPase inhibitors on OSCCs. In this study, the effects of a V-ATPase inhibitor, concanamycin A1 (CMA), on the proliferation and apoptosis of OSCC were investigated *in vitro*. We used four OSCC cell lines, MISK81-5, SAS, HSC-4 and SQUU-B. Acridine orange staining revealed that the red fluorescence was reduced in all of the low concentration CMA-treated OSCC cells, indicating that the acidification of vesicular organelles in the OSCCs was prevented by the treatment with low-concentration of CMA. CMA treatment induced apoptosis in MISK81-5, SAS and HSC-4 cells, but not in SQUU-B cells. The p-p38 expression was not altered in CMA-treated SQUU-B cells, but their levels were increased in the other cells. The Bax/Bcl-2 ratio in CMA-treated SQUU-B cells was dramatically decreased in comparison with that in the other cell lines treated with CMA. However, when the SQUU-B cells were treated with CMA and a histone deacetylase inhibitor, suberoylanilide hydroxamic acid (SAHA), the SQUU-B cells became more susceptible to the CMA-induced apoptosis. SAHA treatment led to a significantly decrease in the Bcl-2 expression in CMA-treated SQUU-B cells, resulting in a dramatically increased Bax/Bcl-2 ratio in comparison with that observed in the SQUU-B cells treated with CMA alone. These findings suggest that CMA could have an anti-tumor effect on OSCCs. In addition, combination of CMA with other agents, such as SAHA, could help improve the pro-apoptotic effects of CMA even in CMA-resistant OSCC cells.

## Introduction

Induction chemotherapy for head and neck cancers reduces the number of patients requiring mandibulectomy and/or radiation therapy. Using it in locally advanced oral cancer can lead to other benefits [1]. Advances in combination treatments have contributed to the improvement of cancer therapy in the last three decades [2]. However, induction chemotherapy has not significantly improved the survival of patients with oral squamous cell carcinoma (OSCC) [1]. Ma et al. [3] reported that there was no significant difference in the overall survival, disease-free survival or locoregional recurrence between the patients treated with and without induction chemotherapy for resectable head and neck squamous cell carcinoma in a meta-analysis of randomized trials (1965–2011). The long-term survival in patients with advanced head and neck squamous cell carcinoma is still poor [2]. Thus, the development of a new chemotherapeutic strategy is required to improve the tumor specificity for OSCC, and to overcome the resistance of such tumors to the chemotherapeutic agents, in order to increase their efficacy and/or to decrease any side effects of the drugs.

OSCCs comprise more than 90% of all malignant epithelial tumors arising in the oral cavity [4], [5]. In solid tumors such as OSCCs, rapid tumor progression is thought to result in hypoxia as the tumor outgrows its vascular supply, which leads to intra- and extracellular acidosis in the tumor microenvironment. This change of the extracellular pH influences several biological behaviors of tumor cells, such as their proliferation [6], invasion and metastasis [7], [8], angiogenesis [9] and drug resistance [10]. The tumor cells must adapt to the decrease in the intracellular pH, because intracellular acidosis allows endonucleases to become activated and induce DNA fragmentation, thus leading to apoptosis [11]–[13].

Accumulating data indicate that vacuolar H^+^-ATPase (V-ATPase) is involved in the acidification of the microenvironment around/in tumors, and that it induces tumor invasion and multi-drug resistance in several malignant tumors [14]–[18]. It has also been reported to help maintain the intracellular pH by extruding protons into the extracellular medium [10], [15], [19]. Highly metastatic cancer cells highly express V-ATPase in the plasma membrane [20], whereas poorly metastatic cells express it at low levels [19]. Highly metastatic cells preferentially use V-ATPase in the plasma membrane to regulate their intracellular alkalosis [19]. Thus, V-ATPase plays a critical role in tumor progression and invasiveness through the creation of the acidic microenvironments.

In addition, the gene encoding the V-ATPase subunit C is overexpressed in multidrug-resistant HL60 cells [21]. Murakami et al. [22] also found overexpression of the V-ATPase subunit C (ATP6C) gene in cisplatin-resistant tumors. A combination of cisplatin and bafilomycin, a V-ATPase inhibitor, was enhanced the cytotoxicity in cisplatin-resistant cells. Chauhan et al. [23] suggested that an endosomal acidification is involved in the cisplatin resistance of adenocarcinoma cell lines. The use of V-ATPase inhibitors in combination therapy improves the cytotoxicity of other drugs, such as camptothecins and oxaliplatin [24], [25]. These results indicated that V-ATPase inhibitors have the potential to increase the tumor sensitivity to chemotherapeutic drugs.

Recently, Otero-Rey et al. [26] reported that OSCCs most strongly express the ATP6V1C1 gene, compared to other genes of the V-ATPase complex, by a cDNA array analysis. This suggests that the OSCCs increase the V-ATPase activity by inducing its overexpression to change the intra- and extracellular pH. Immunohistochemically, the OSCCs showed more intense staining for ATP6V1C1 compared to the normal oral mucosal counterparts [27]. Because the inhibition of V-ATPase has been proposed to exert similar effects on the growth and apoptosis of OSCCs as it did against other carcinomas, targeting V-ATPase in tumors may provide a new therapeutic intervention for OSCCs. However, there is little information available regarding the effects of V-ATPase inhibitors on OSCCs, and much less on the difference in the signaling pathways between the V-ATPase inhibitor-resistant and sensitive OSCCs treated with the V-ATPase inhibitor.

In this study, the effects of a V-ATPase inhibitor, concanamycin A1 (CMA), on the proliferation and apoptosis of OSCC were investigated *in vitro*. Treatment with a low-concentration of CMA prevented the acidification of vesicular organelles in the MISK81-5, SAS, HSC-4 and SQUU-B OSCC cells used in this study. However, while CMA induced apoptosis in MISK81-5, SAS and HSC-4 cells, it did not in SQUU-B cells. We demonstrated that suberoylanilide hydroxamic acid (SAHA), a histone deacetylase inhibitor (HDACi), led to an increased susceptibility of the SQUU-B cells to CMA-induced apoptosis. These findings suggest that CMA may be a candidate therapeutic agent against OSCCs, and that combination therapy using CMA with other agents, such as SAHA, could improve the efficacy of CMA treatment, allowing apoptosis to be induced even in the CMA-resistant OSCC cells.

## Materials and Methods

### Reagents

CMA, a V-ATPase inhibitor, was purchased from WAKO (Osaka, Japan). Suberoylanilide hydroxamic acid (SAHA), a histone deacetylase, was purchased from Cayman Chemical (Ann Arbor, MI, USA). HA14-1 (Ethyl [2-amino-6-bromo-4-(1-cyano-2-ethoxy-2-oxoethyl)]-4H-chromene-3-carboxylate, 2 Amino-6-bromo-α-cyano-3-(ethoxycarbonyl)-4H-1-benzopyran-4-acetic acid ethyl ester), a small molecule inhibitor of Bcl-2, was obtained from Sigma (St. Louis,MO, USA). All other chemicals and reagents were purchased from Life Technologies (Carlsbad, CA) or Sigma (St. Louis, MO, USA), unless otherwise specified.

### Cell lines and culture conditions

Four human OSCC cell lines, MISK81-5 [28], HSC-4 [29], SAS [30] and SQUU-B [31], were used in the study. A human keratinocyte cell line, HaCaT [32], was also used. The MISK81-5 cells were grown in α-MEM (Life Technologies) supplemented with 10% fetal bovine serum. The HSC-4 and HaCaT cells were cultured in D-MEM (Life Technologies) supplemented with 10% fetal bovine serum. The SAS and SQUU-B cells were maintained in DMEM/F-12 (Life Technologies) supplemented with 10% fetal bovine serum. These cell lines were maintained in the medium with 10% fetal bovine serum, 100 IU/ml penicillinand 100 µg/ml streptomycin (Life Technologies). The medium supplemented with the indicated components was used in the following analyses, such as a cell proliferation and cell death assay.

### Acridine orange staining of intracellular acidic organelles

Cells were treated with 2.5 nM CMA for 2 hr, washed with PBS three times and stained with acridine orange at a final concentration of 1.25 µg/ml for 15 min. The fluorescent images were observed under a fluorescent microscope equipped with a mirror unit U-MWB2 and Microscope Digital Camera System DP72 (Olympus, Tokyo, Japan).

### Observations of morphological changes

The morphological changes of the MISK81-5, SAS, HSC-4 and SQUU-B cells were observed under a phase contrast microscope 24 hr after the treatment.

### Cell proliferation

The cell proliferation was assessed on the cell viability measured by the MTS [3-(4,5-dimethyl-2-yl)-5-(3-carboxymethoxyphenyl)-2-(4-sulfophenyl)-2H-tetrazolium, inner salt] assay. MISK81-5 and HSC-4 cells, and SAS and SQUU-B cells, were plated at a density of 7500 and 5000 cells per well in 96-well plates, respectively. The cells were treated in serum-free medium containing the indicated concentrations of CMA for 48 hr. Then, 20 µl of CellTiter 96 AQueous One Solution Reagent (Promega, Madison, WI, USA) was added into each well of the 96-well plates. The cells were incubated for 2 hr at 37°C, then the absorbance at 490 nm was measured using an Infinite M200 spectrometer (TECAN, Sunrise, Switzerland).

### DNA fragmentation

MISK81-5, SAS, HSC-4 and SQUU-B cells were plated in 100 mm dishes at a density of 0.75**×**10^5^ cells/ml, and were cultured for 24 hr. Then, after 24 hr of treatment with 2.5 nM CMA or with the vehicle, genomic DNA was extracted from each cell line using the Wizard SV Genomic DNA kit (Promega) according to the manufacturer's instructions. Equal amounts (5 µg) of the extracted DNA were electrophoresed on 1.9% agarose gels, and the gels were then stained with ethidium bromide for visualization of the DNA by a UV light.

### Cell death assay

The cells were seeded on 8-well chamber slides (1.25**×**10^4^ cells/well). After 48 hr, the culture medium was changed to a serum-free medium with 0 or 2.5 nM of CMA. The cells were then incubated for an additional 24 hr. The TdT-mediated dUTP-biotin nick labeling (TUNEL) method was applied as a cell death assay using the *in situ* Apoptosis Detection Kit (Takara, Shiga, Japan). Briefly, TdT enzymes were reacted with the cells cultured on the chamber slides, then the cells were labeled with FITC on the nick sites of the DNA. DAPI was used for nuclear staining. The numbers of DAPI-stained cells and TUNEL-positive cells were counted in six different microscopic fields of each well. More than 3,300 DAPI-stained cells were examined in each well. The number of TUNEL-positive cells was divided by that of DAPI-stained cells to calculate the ratio of TUNEL staining. At least three independent experiments were performed in triplicate.

### qRT-PCR

Total RNA was isolated from the cells using the SV total RNA isolation system (Promega). The cDNA was prepared by a reverse transcription reaction using the SuperScript VILO cDNA synthesis system (Life Technologies). Real-time quantitative reverse transcription PCR (qRT-PCR) was performed with a SYBR Premix Ex Taq II kit (Takara) according to the manufacturer's instructions. Glyceraldehyde-3-phosphate dehydrogenase (GAPDH) was used as an internal control. The specific primer sets used were as follows: ATP6V0A3 (TCIRG1), 5′-GGC CAC GGG CTG CTC ATG TT-3′ and 5′-CTG GTG GCG CGA CTG AAG CA-3′; ATP6V0A4, 5′-TTC AGA CGC GAG GCT GGG GA-3′ and 5′-GTC GCA GGG CGT GCA GGA AA-3′; ATP6V1C1, 5′-GCA TGC GGC AAC TTC AAA GA-3′ and 5′-GCC AAC CAA GAC ATC CAA CG-3′; Bax, 5′-GGC CGG GTT GTC GCC CTT TT-3′ and 5′-CCG CTC CCG GAG GAA GTC CA-3′; Bcl-2, 5′-GAA CCG GCA CCT GCA CAC CTG-3′ and 5′-AAG CTC CCA CCA GGG CCA AA-3′; GAPDH, 5′-GCA CCG TCA AGG CTG AGA AC-3′ and 5′-TGG TGA AGA CGC CAG TGG A-3′. A melting curve analysis and gel electrophoresis of the PCR products were used to confirm the amplification specificity of each primer set. All mRNA expression levels were normalized to that of GAPDH. All experiments were performed in triplicate.

### Western blot analyses of proteins related to MAPK and apoptosis

MISK81-5, SAS, HSC-4 and SQUU-B cells were lysed in lysis buffer [20 mM Tris-HCl (pH 7.5), 150 mM sodium chloride, 2.5 mM sodium pyrophosphate, 0.1% SDS, 1 mM EDTA, 1 mM EGTA and 1% Triton X-100] containing proteinase and phosphatase inhibitors [1 mM phenylmethylsulfonyl fluoride, 1 mM β-glycerophaspahate, 1 mM Na_3_VO_4_ and 4% protease inhibitor cocktail]. The protein concentration was quantified using a Micro BCA Protein Assay Kit (Thermo, Rockford, IL, USA). The protein samples (30 or 40 µg/lane) were separated by 10 or 12% SDS–PAGE, and were electrotransferred to Immun-Blot PVDF membranes (Bio-Rad, Hercules, CA, USA). The membranes were probed with primary antibodies for 1 hr and incubated for 1 hr with secondary antibodies conjugated with peroxidase (GE, Buckingham, UK). The primary antibodies against Bax and Bcl-2 were incubated with the membrane overnight at 4°C. Chemiluminescent signals were then developed with ECL Prime (GE) and detected using a cooled CCD-camera (LAS-1000, Fujifilm, Tokyo, Japan). Antibodies for phospho-p38 MAPK (pT180/pY182), p38α(SAPK2a), phospho-STAT3 (pY705, pS727) and total STAT3 were purchased from BD Biosciences (Franklin Lakes, NJ, USA). Antibodies against Bax and Bcl-2 were obtained from Cell Signaling Technology (Danvers, MA, USA). The antibody for β-actin was purchased from Sigma (St. Louis, MO, USA). The procedure was identical to those used in previous study [33]. In the semiquantitative analyses of the levels of protein expression, the intensity of the bands was measured using the “ImageJ” (Image J ver. 1.44, http://rsb.info.nih.gov/ij/index.html) densitometric analysis software program. β-actin was used as an internal control protein. The target protein/β-actin ratio based on the intensity of the bands was calculated [34].

### CMA-induced cytotoxicity in cells treated with siRNA against Bcl-2 in cell culture

According to the manufacturer's protocol, Bcl-2-siRNA (final conc. 20 nM) was transfected into SQUU-B cells using Lipofectamine RNAiMAX (Invitrogen) and Opti-MEM (Invitrogen) [33] in 96-well plates at 24 hr after cell seeding. siRNA for human Bcl-2 (Hs_BCL2_2981) and a universal negative control siRNA (Sigma) were used as a target and negative control, respectively. At 24 hr after transfection with siRNA, the cells were treated in serum-free medium containing the indicated concentrations of CMA for 48 hr. Then, the MTS assay was performed.

### Statistical analyses

All experiments were independently repeated at least three times. The results are expressed as the means ± SEM. The statistical analyses were performed using a one-way ANOVA with the Tukey-Kramer comparison test, Dunnett's test, and using Student's *t*-test. Differences resulting in a *p*-value <0.05 or 0.01 were considered to be statistically significant.

## Results

### Concanamycin A inhibited the acidification of vesicular organelles and the proliferation of OSCC cells

**Figure 1:**
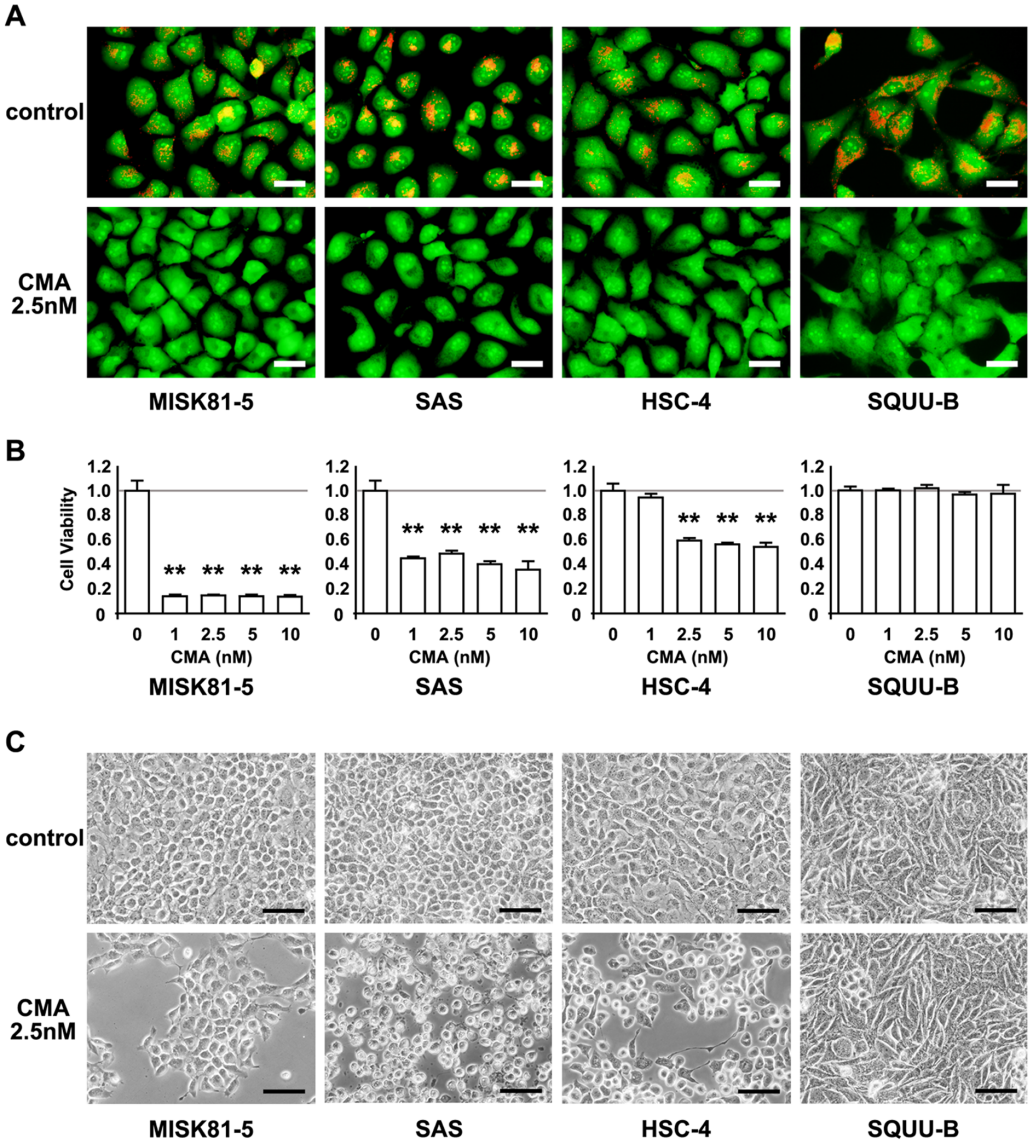
Effects of CMA on the acidification of vesicular organelles and the viability of OSCC cells. **A**. Acridine orange labeling of acidic vesicles in OSCC cells in the presence or absence of CMA (2.5 nM). The acidic vesicles show orange-red fluorescence, whereas the cytoplasm and nucleus show green fluorescence. CMA treatment suppressed the acridine orange staining of the acidic vesicles in OSCC cells. Scale bar; 30 µm. **B**. OSCC cells were incubated in α-MEM, DMEM or DMEM/F-12 medium in the presence of CMA at the indicated concentrations for 48 hr. The cell viability was determined by the MTS assay as described in the Materials and methods. *Columns*, means of at least triplicate experiments; bars, SD. *p<0.05 and **p<0.01 versus the untreated cells. **C**. The number of cells remaining on the dish and morphological changes of OSCC cells were observed by phase contrast microscopy. At 24 hr of treatment, a decreased number of cells and morphological changes were noted in the CMA-sensitive cells (MISK81-5, HSC-4 and SAS cells) compared with the untreated cells, whereas little change was observed in the CMA-resistant SQUU-B cells in comparison to the untreated cells. Scale bar; 80 µm.

To assess the effects of CMA on the V-ATPase inhibition in OSCC cells, the acidic vesicular organelles in these carcinoma cells were visualized by acridine orange staining. In the stained cells, acidic vesicles including lysosomes fluoresced bright red, whereas the cytoplasm and nucleus fluoresced green. Staining of vital MISK81-5, SAS, HSC-4 and SQUU-B cells with acridine orange revealed that the red fluorescence completely disappeared after treatment with 2.5 nM CMA for 2 hr (Fig. 1A), indicating that CMA inhibited the acidification of vesicular organelles in the OSCC cells. Conversely, red fluorescent organelles were observed in the untreated cells used as a control.

To evaluate the effects of V-ATPase inhibition by CMA on the proliferation of the OSCC cells, we performed the MTS assay in MISK81-5, SAS, HSC-4 and SQUU-B cells in the presence and absence of 0, 1, 2.5, 5 or 10 nM CMA for 48 hr. As shown in Fig. 1B, CMA inhibited the growth of the MISK81-5, SAS and HSC-4 cells, showing that these cells were sensitive to CMA. The viability of the MISK81-5 and SAS cells was decreased by about 14 and 45%, respectively, after 1 nM CMA treatment compared to that observed in the untreated cells. In contrast, the SQUU-B cells were resistant to CMA treatment at even the highest concentration (10 nM).

By phase contrast microscopy, morphological changes were observed in some of the CMA-treated MISK81-5, SAS and HSC-4 cells (Fig. 1C). At 24 hr after the addition of CMA, the MISK81-5, SAS and HSC-4 cells were observed to be detached from the culture dish, while a decreased number of cells remained on the culture dish, and these cells exhibited substantial morphological changes. In contrast, the morphology of SQUU-B cells remained largely unchanged, and there was no apparent decrease in the number of the cells remaining on the culture dish after the CMA treatment.

### Concanamycin A induced apoptosis in OSCC cells

**Figure 2:**
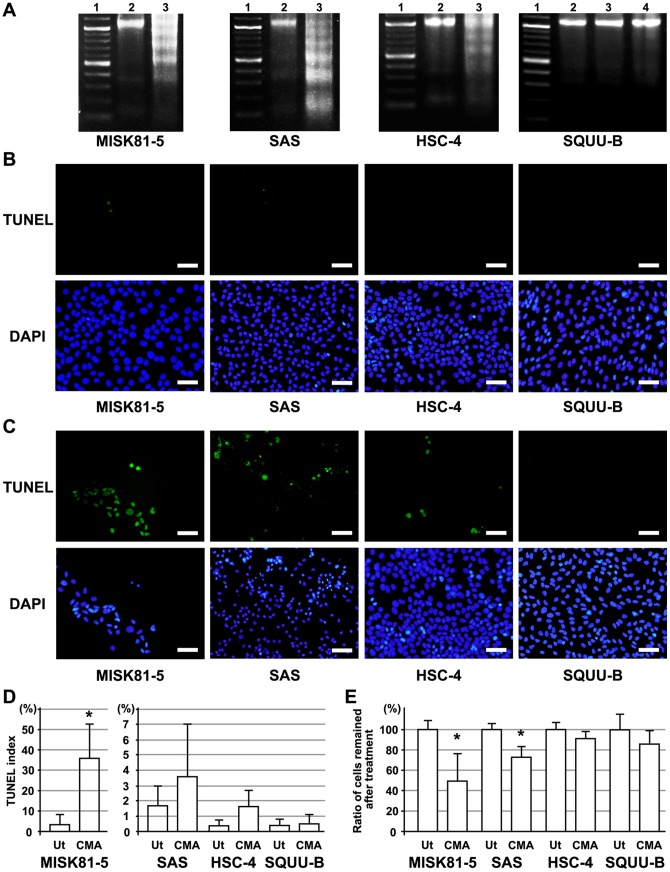
CMA-induced apoptosis in OSCC cells. **A**. Nucleosomal DNA fragmentation was seen in MISK81-5, SAS and HSC-4 cells after CMA treatment. In contrast, little DNA fragmentation was found in SQUU-B cells after treatment with 2.5 or 5 nM CMA. Lane 1, 100bp ladder marker; Lane 2, untreated control; lane 3, exposed to 2.5 nM CMA for 24 hr; Lane 4, exposed to 5 nM CMA for 24 hr (only in SQUU-B cells). **B**. Few TUNEL-positive cells were observed in the untreated MISK81-5, SAS, HSC-4 and SQUU-B cells. DAPI-stained nuclei indicated the cells remaining on the culture slide. Scale bar; 60 µm. **C**. TUNEL-positive cells were observed in the MISK81-5, SAS and HSC-4 cells treated with CMA for 24 hr. However, few TUNEL-positive SQUU-B cells were seen. Scale bar; 60 µm. **D**. The TUNEL index was calculated as the ratio of TUNEL-positive cells observed in the remaining DAPI-positive cells. Ut: untreated cells, CMA: cells treated with 2.5 nM CMA. *p<0.05 versus the untreated cells. **E**. The number of DAPI-positive cells remaining on the slide observed in the CMA-treated cells was compared with that observed in the untreated cells. For untreated cells, at least 3,300 DAPI-positive cells were counted in each experiment. The DAPI-positive cells in the CMA-treated cells were counted in the same area. Ut: untreated cells, CMA: cells treated with 2.5 nM CMA. *Columns*, means of at least triplicate experiments; bars, SD. *p<0.05 versus the untreated cells.

To confirm the pro-apoptotic effects of CMA on the OSCC cells, the changes in apoptotic cell death were confirmed by detecting DNA fragmentation and performing the TUNEL assay. After MISK81-5, SAS, HSC-4 and SQUU-B cells were treated with 2.5 nM CMA for 24 hr, the genomic DNA was extracted from the treated cells for gel electrophoresis. As shown in Fig. 2A, the CMA treatment substantially induced DNA ladder formation in the MISK81-5, SAS and HSC-4 cells. In contrast, the SQUU-B cells showed minimal DNA fragmentation following treatment with CMA.

TUNEL-positive nuclei were observed in the MISK81-5, SAS and HSC-4 cells 24 hr after the treatment with 2.5 nM CMA (Fig. 2B, C). However, little fluorescence was observed in the SQUU-B cells (Fig. 2B, C). The TUNEL index was calculated by dividing the number of TUNEL-positive cells by the number of the cells remaining on the dish. The TUNEL index in the MISK81-5 cells was significantly increased by about 10-fold after CMA treatment compared to the control. The TUNEL index in the HSC-4 and SAS cells was increased by about 2- and 4-fold after CMA treatment, respectively, compared to the control cells (Fig. 2D). However, these differences were not significant. Meanwhile, the TUNEL indices in the treated and untreated SQUU-B cells were less than 0.5%, which was not significantly different from the control.

After the CMA treatment, the number of MISK81-5 and SAS cells remaining on the culture dishes was significantly decreased, by 49% and 73%, compared to the untreated cells (Fig. 2E). Meanwhile, the number of remaining treated HSC-4 and SQUU-B cells were slightly decreased compared with that observed in the untreated cells, but there were no significant differences between the control and treated cells (Fig. 2E).

### Comparison of the gene expression of subunits of V-ATPase, Bax and Bcl-2 in OSCC cells without CMA treatment

In order to make comparisons between the cell lines with sensitivity to CMA and the SQUU-B cells, which have CMA-resistance, an analysis of the V-ATPase subunit gene expression of the a3 and a4 isoforms in a subunit of the V0 transmembrane domain (ATP6V0A3 and ATP6V0A4) and C1 subunit of the V1 intra-membrane domain (ATP6V1C1) of V-ATPase was performed without CMA treatment. The V0 a subunit is the largest of the V0 subunits and contains four isoforms, namely a1, a2, a3 and a4 [35]. Several previous reports focused on the expression of ATP6V0A3, ATP6V0A4 and ATP6V1C1, which are associated with malignant cell behavior and a malignant phenotype [18], [26], [36]. HaCaT cells, a human keratinocyte cell line, were used as a control.

**Figure 3:**
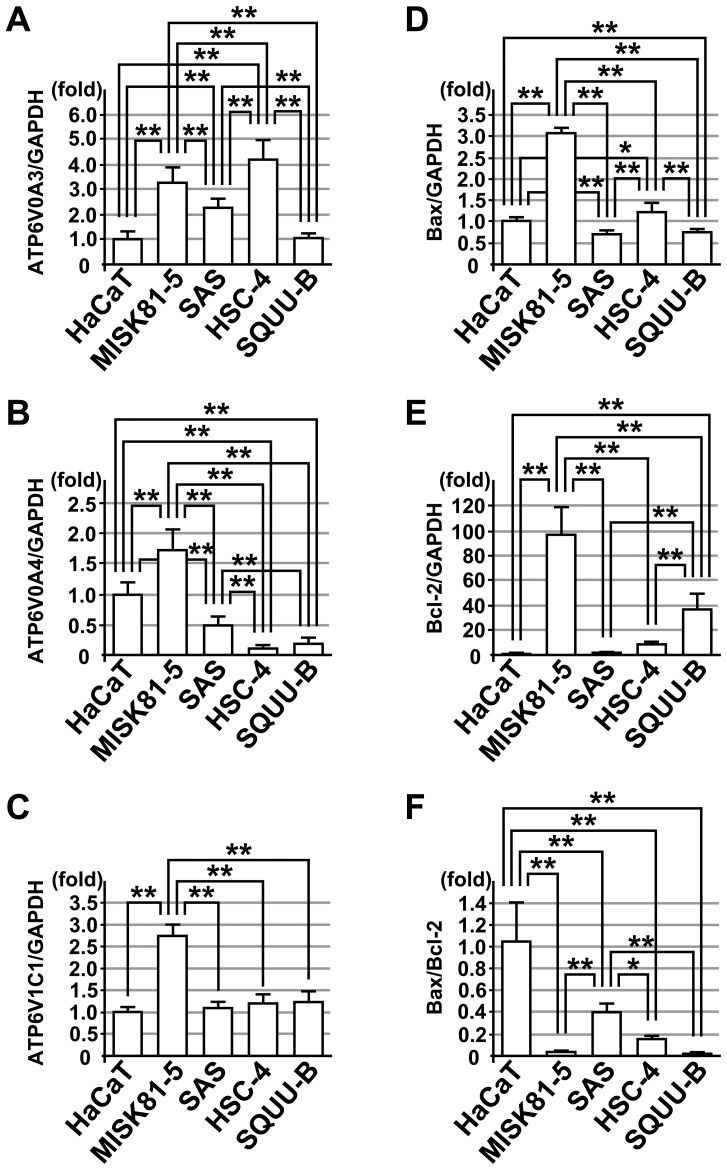
Expression of V-ATPase subunit genes and pro- and anti-apoptotic genes in OSCC cells. Expression of V-ATPase subunit genes and pro- and anti-apoptotic genes was compared in OSCC cell lines, MISK81-5, SAS, HSC-4 and SQUU-B cells, without CMA treatment. The ATP6V0A3 (**A**) ATP6V0A4 (**B**) and ATP6V1C1 (**C**) mRNA expression levels were analyzed by qRT-PCR. The Bax (**D**) and Bcl-2 (**E**) mRNA expression levels were also analyzed. The Bax/Bcl-2 ratio (**F**) in each OSCC cell line was calculated based on the qRT-PCR data. GAPDH was used as an internal control for the experiment. *Columns*, means of at least triplicate experiments; bars, SD. * and ** denote *p*<0.05 and *p*<0.01 compared to the untreated cells, respectively.

The MISK81-5 cells showed significantly higher expression of the ATP6V0A3, ATP6V0A4 and ATP6V1C1 genes than to that did the HaCaT cells (Fig. 3A-C). Although the expression levels of ATP6V0A3 in the SAS and HSC-4 cells were significantly higher than that in the HaCaT cells, that in the SQUU-B cells was similar to that in the HaCaT cells (Fig. 3D). The ATP6V0A4 mRNA expression in the SAS, HSC-4 and SQUU-B cells was less than 50% of that in the HaCaT cells, and was significantly lower than that of the HaCaT cells (Fig. 3E). Although the ATP6V1C1 expression in the SAS, HSC-4 and SQUU-B cells were slightly higher than that of the HaCaT cells, there was not significant difference among the cells.

Next, because Bcl-2 is occasionally associated with resistance to anti-tumor agents, and is often observed in human carcinomas [37], we compared the expression of Bax (pro-apoptotic) and Bcl-2 (anti-apoptotic) mRNAs, which are parts of the mitochondrial pathway, in the OSCC cells in the absence of CMA.

The Bax mRNA expression in the MISK81-5 cells was three-fold higher than that in the HaCaT cells, and was significantly higher than that of the other carcinoma cells examined. The expression in the SAS and SQUU-B cells was significantly lower than that in the HaCaT cells (Fig. 3D). The Bcl-2 mRNA expression in the MISK81-5 and SQUU-B cells was markedly higher than that of the other cells, and approximately one hundred-fold and forty-fold compared to that in the HaCaT cells, respectively (Fig. 3E). In the SAS and HSC-4 cells, the expression level was slightly higher than that of the HaCaT cells. The Bax/Bcl-2 ratios in all the carcinoma cells examined were less than 0.4, and were significantly lower than that of the HaCaT cells (*p*<0.01)(Fig. 3F). Although the Bax/Bcl-2 ratios in the MISK81-5 and SQUU-B cells were markedly low, that of the SQUU-B cells was slightly lower than that of the MISK81-5 cells.

### CMA increased the Bax/Bcl-2 ratio in CMA-sensitive OSCC cells

Because the release of cytochrome c from the mitochondria into the cytoplasm was involved in the apoptotic cell death induced by concanamycin A [38], we evaluated the expression of Bax and Bcl-2 mRNAs in the OSCC cells in presence and absence of CMA by qRT-PCR.

**Figure 4:**
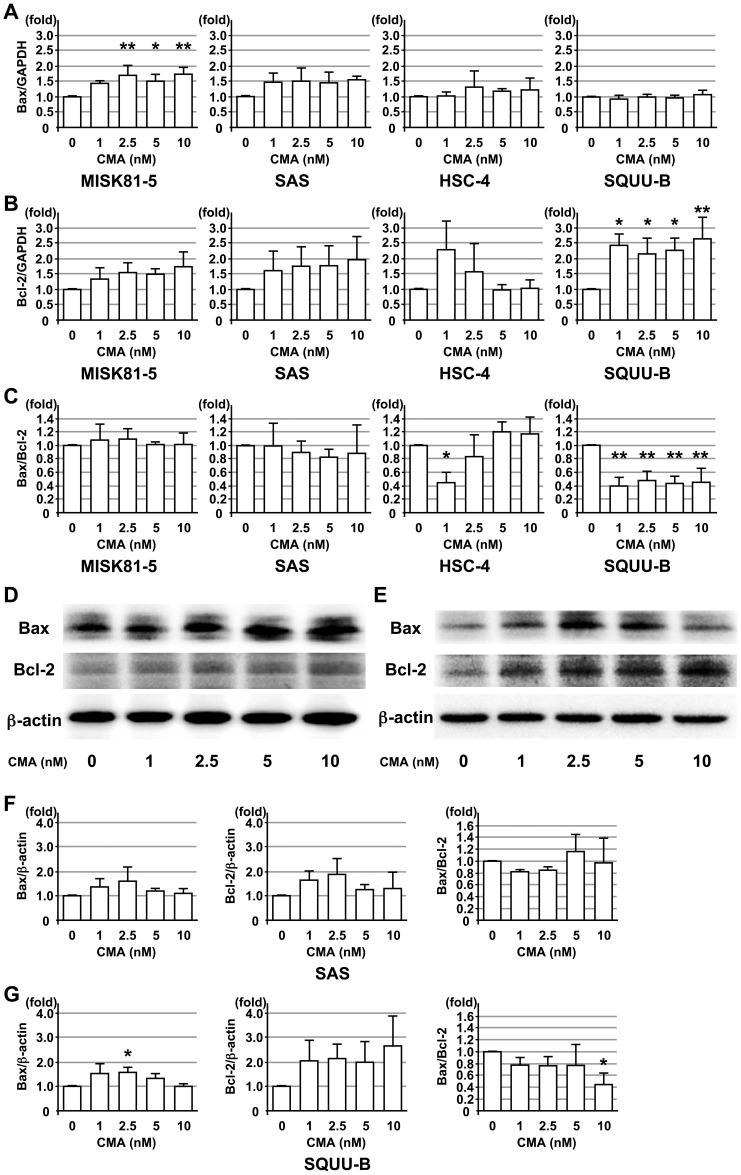
Effects of CMA on the expression of pro- and anti-apoptotic genes and proteins in OSCC cells. OSCC cell lines, MISK81-5, SAS, HSC-4 and SQUU-B cells, were treated with or without CMA. The Bax (**A**) and Bcl-2 (**B**) mRNA expression levels were analyzed at 24 hr after CMA treatment by qRT-PCR. The Bax/Bcl-2 ratio (**C**) in each OSCC cell line was calculated based on the qRT-PCR data. GAPDH was used as internal control for the experiment. The Bax and Bcl-2 protein expression levels in the SAS (**D**) and SQUU-B (**E**) cell lines were analyzed at 24 hr by a Western blot analysis. The Bax level, Bcl-2 level and Bax/Bcl-2 ratio was calculated based on the intensity of the bands in the SAS (**F**) and SQUU-B (**G**) cell lines. β-actin was used as an internal control for the experiment. *Columns*, means of at least triplicate experiments; bars, SD. * and ** denote *p*<0.05 and *p*<0.01 compared to the untreated cells, respectively.

The CMA treatment of MISK81-5 and SAS cells upregulated the mRNA expression of both Bax and Bcl-2 after 24 hr of treatment compared to the untreated cells (Fig. 4A, B). In the HSC-4 cells, CMA treatment slightly upregulated the mRNA expression of Bax. Although the treatment with 1 and 2.5 nM of CMA upregulated the mRNA expression of Bcl-2, the treatment with 5 and 10 nM CMA treatments did not induce any significant changes compared to the levels in the untreated cells (Fig. 4B).

Interestingly, CMA treatment of the SQUU-B cells resulted in an increase in the mRNA expression of Bcl-2 by more than two-fold (Fig. 4B), but there was little change in the Bax mRNA expression compared to the untreated cells (Fig. 4A). Accordingly, the Bax/Bcl-2 ratios in the SQUU-B cells were significantly decreased by the CMA treatment at all of the concentrations examined in this study (Fig. 4C).

Immunoblotting revealed that SAS cells had upregulated the protein expression of both Bax and Bcl-2 after 24 hr of treatment compared to the untreated cells (Fig. 4D, F). The Bax/Bcl-2 ratios in the SAS cells were similar to that of the mRNA expression (Fig. 4F). On the other hand, although the Bax protein expression in the CMA-treated SQUU-B cells was slightly upregulated compared to the untreated cells, CMA treatment of the SQUU-B cells resulted in an increase in the protein expression of Bcl-2 by more than two-fold (Fig. 4E, G). The Bax/Bcl-2 ratios in the SQUU-B cells were decreased by the CMA treatment (Fig. 4G).

### Concanamycin A induced the phosphorylation of p38 in CMA-sensitive OSCC cells

It was previously reported that p38, one of the MAPKs was activated during the induction of apoptosis by a V-ATPase inhibitor, and that it was involved in the anti-mitogenic action of the inhibitor [39]–[41]. We therefore examined the phosphorylation of p38 during CMA-induced apoptosis by immunoblotting.

**Figure 5:**
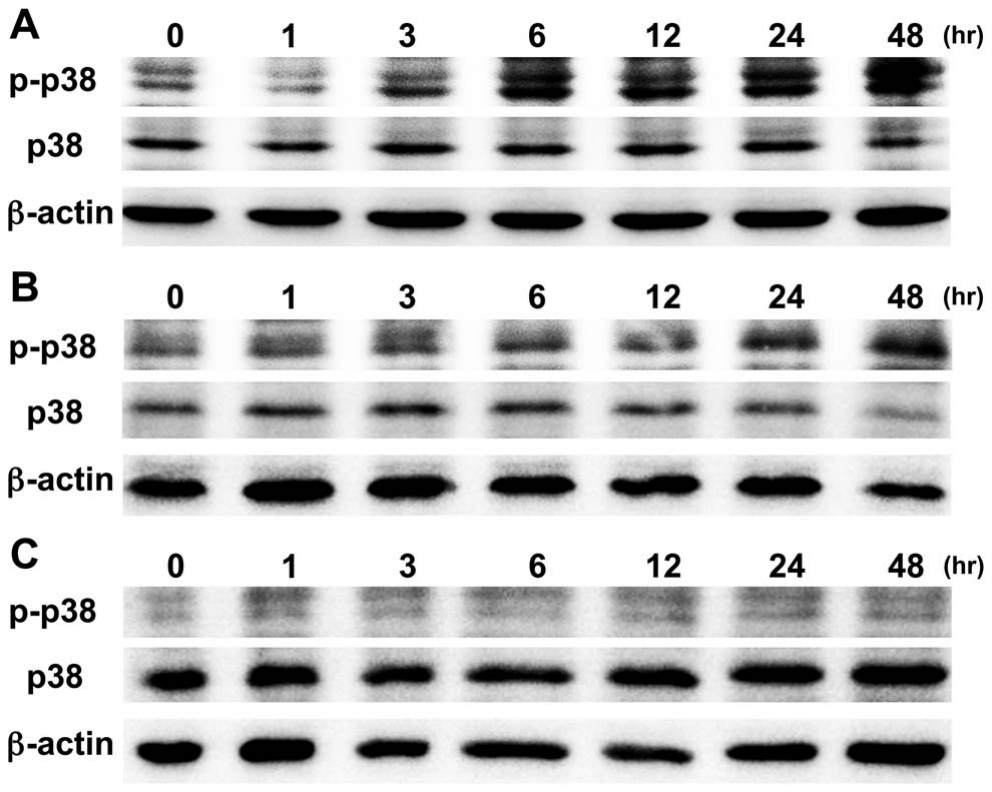
The involvement of p38 in apoptosis of OSCC cells induced by CMA. CMA (2.5 nM) time-dependently increased the phosphorylation of p38 in CMA-sensitive MISK81-5 (**A**) and SAS (**B**) cells. On the other hand, CMA-resistant SQUU-B (**C**) cells showed little change in p38 phosphorylation. The protein samples were normalized to the level of β-actin.

Our results revealed that the phosphorylation of p38 was upregulated in MISK81-5 cells from 6 to 48 hr after beginning the treatment with CMA (Fig. 5A). The phosphorylation of p38 in the SAS cells was observed at 24 and 48 hr after treatment with CMA (Fig. 5B). In contrast, the phosphorylation of p38 was only minimally changed in the SQUU-B cells at the time points examined (Fig. 5C).

### The apoptosis in CMA-resistant OSCC cells was enhanced by concurrent treatment with SAHA and CMA

To the best of our knowledge, there is little information available regarding the combination treatment for CMA-resistant OSCC cells. Concurrent treatment of cells with SAHA enhanced the cisplatin-induced apoptosis in cisplatin-resistant OSCC cells [42]–[44]. Therefore, MTS assays were carried out to compare the cell viabilities of SQUU-B cells treated with SAHA, CMA, or a combination of the agents, and to evaluate whether SAHA can increase the CMA-induced cytotoxicity.

**Figure 6:**
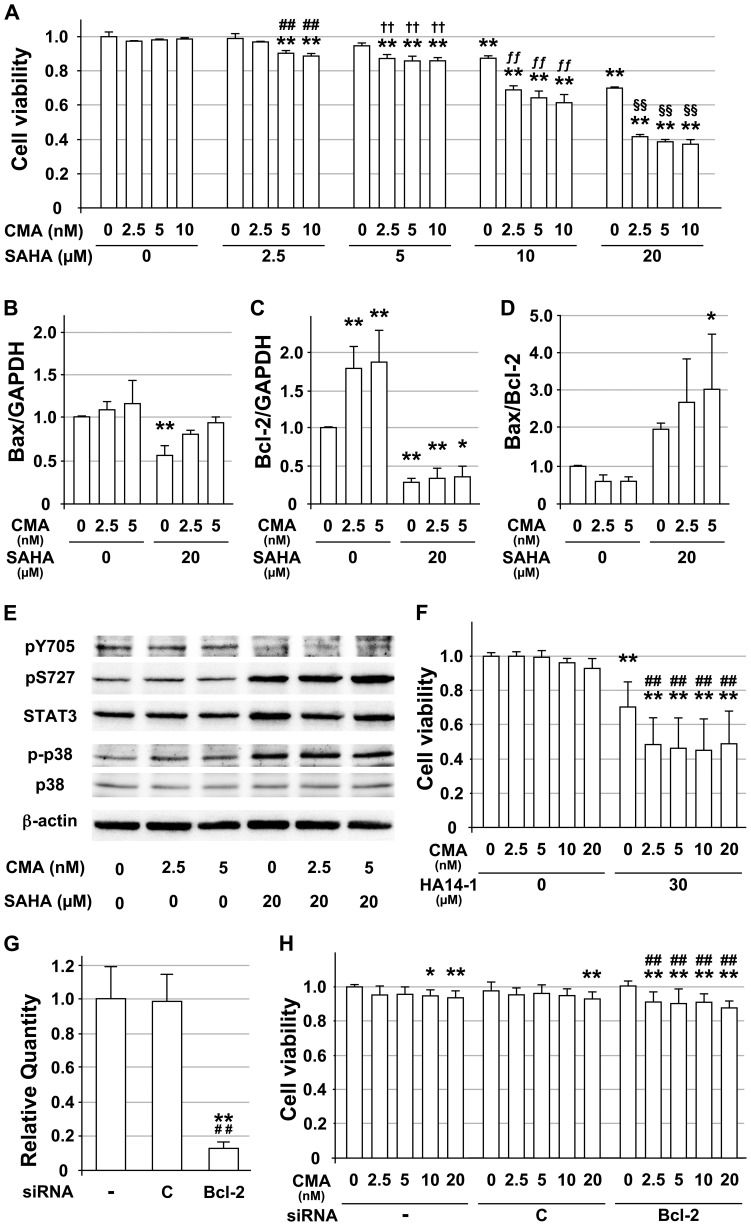
Effects of concurrent treatment with SAHA and CMA on the CMA-resistant OSCC cells. **A**. The MTS assay was performed on cells treated with SAHA, CMA, or the combination of these two agents, for 48 hr. The cell viability was normalized to that of the untreated cells. ** denotes *p*<0.01 compared to the untreated cells. ## denotes *p*<0.01 compared to the cells treated with SAHA alone at the indicated concentration. ††, ƒƒ and §§ also indicate *p*<0.01 compared to the cells treated with SAHA alone at the indicated concentration. **B**. The Bax expression in SQUU-B cells treated with SAHA (20 µM), CMA (2.5 or 5 nM), or the combination of these two agents for 24 hr is shown. The data were normalized to the level of GAPDH. ** denotes *p*<0.01 compared to the untreated cells. **C**. The Bcl-2 expression in the same samples. The data were normalized to that of GAPDH. * and ** denote *p*<0.05 and *p*<0.01 compared to the untreated cells, respectively. **D**. The relative fold-change in the mRNA expression of Bax and Bcl-2 based on the data in Figs. 6B and C. *Columns*, means of at least triplicate experiments; bars, SD. * denotes *p*<0.05 compared to the untreated cells. **E**. The cells were treated with SAHA, CMA, or the combination of these two agents for 24 hr, and then the proteins were extracted for an immunoblot analysis. The immunoblots were probed using antibodies against STAT3, STAT3 phosphorylated on Y705 or S727, and phosphorylated mitogen-activated protein kinase (MAPK), as indicated. The protein levels were normalized to the level of β-actin. **F**. Cells were pre-treated with or without 30 µM HA14-1 for 6 hr, followed by a 48 hr treatment with CMA at the indicated concentrations. ** denotes *p*<0.01 compared to the untreated cells, respectively. ## denotes *p*<0.01 compared to the cells treated with HA14-1 alone at the indicated concentration. *Columns*, means of at least triplicate experiments; bars, SD. **G**. siRNA selectively reduced the gene expression in the SQUU-B cells 24 hr after siRNA transfection. ** denotes *p*<0.01 compared to the untreated cells (-). ## denotes *p*<0.01 compared to the cells treated with negative control siRNA (C). GAPDH was used as a reference gene. **H**. Cells were pre-treated with or without 20 nM Bcl-2-siRNA for 24 hr, followed by a 48 hr treatment with CMA at the indicated concentrations. * and ** denote *p*<0.05 and *p*<0.01 compared to the untreated cells, respectively. # and ## denote *p*<0.05 and *p*<0.01 compared to the cells treated with Bcl-2-siRNA alone at the indicated concentration. -, sample without siRNA treatment; C, sample treated with negative control siRNA; Bcl-2, sample treated with siRNA for Bcl-2. *Columns*, means of at least triplicate experiments; bars, SD.

The MTS assay revealed that combination treatment with both CMA and SAHA enhanced the CMA-induced cytotoxicity. The cell viability following the administration of almost all the combination treatments significantly decreased compared to the cell viability observed in the untreated cells. These combination treatments led to a significant decrease in the cell viability compared to that observed in the cells treated with CMA or SAHA alone (*p*<0.01) (Fig. 6A). As noted above, CMA treatment did not induce apoptosis in the SQUU-B cells, but treatment with 10 and 20 µM SAHA alone had mild and moderate apoptotic effects on SQUU-B cells, respectively.

To assess the effects of combination treatment with CMA and SAHA on the death of the CMA-resistant SQUU-B cells, the expression levels of Bax and Bcl-2 mRNAs were examined by qRT-PCR. Although SAHA treatment significantly reduced the levels of the pro-apoptotic Bax by about 55% compared to that observed in the untreated cells, the combination treatment recovered the level of the Bax mRNA expression so that it was almost equal to that in the untreated cells (Fig. 6B).

Interestingly, SAHA treatment dramatically reduced the levels of the mRNA for anti-apoptotic Bcl-2. Compared to that observed in the untreated cells, the mRNA expression of Bcl-2 was significantly decreased by about one-third following treatment with SAHA (Fig. 6C), whereas after single treatment with 2.5 nM or 5 nM CMA, the Bcl-2 mRNA expression increased by 1.8-fold (*p* **<**0.01) and 1.9-fold (*p* **<**0.01), respectively. As a result, the combination treatment significantly increased the Bax/Bcl-2 ratio by more than 2.5-fold compared with that observed in the untreated cells (Fig. 6D).

Because Bcl-2 is downstream of STAT3, the phosphorylation of STAT3 and p38 in the SQUU-B cells treated with both CMA and SAHA was examined by immunoblotting (Fig. 6E). The tyrosine phosphorylation of STAT3 (pY705-STAT3) decreased in the SQUU-B cells treated with SAHA alone or both CMA and SAHA, compared that in the SQUU-B cells treated with CMA alone. Meanwhile, the serine phosphorylation of STAT3 (pS727-STAT3) increased in the SAHA-treated SQUU-B cells compared that in the SQUU-B cells treated with CMA alone. SAHA treatment also upregulated the protein expression level of STAT3 in the SQUU-B cells. Additionally, the phosphorylation of p38 was upregulated in SQUU-B cells after 24 hr of treatment with SAHA.

Based on these findings, we next investigated the effect of a Bcl-2 inhibitor, HA14-1, on the viability of SQUU-B cells (Fig. 6F). Although the viability of SQUU-B cells subjected to HA14-1 monotherapy was significantly decreased, that of the cells treated with the combination of CMA and HA14-1 was more strongly decreased, thus indicating that this combination also led to enhanced apoptosis.

In addition, to confirm whether the Bcl-2 inhibition induced apoptosis in the SQUUB cells treated with CMA, we used siRNA to selectively reduce the Bcl-2 expression. Treatment with the siRNA for Bcl-2 induced a significant downregulation of the Bcl-2 mRNA expression (Fig. 6G). The viability of the SQUU-B cells pretreated with siRNA for Bcl-2 was more strongly decreased in response to CMA (Fig. 6H). The effects of HA14-1 monotherapy and Bcl-2 inhibition using siRNA suggested that the downregulation of Bcl-2 is at least partly responsible for the increased apoptosis induced by the combination of CMA and SAHA.

## Discussion

It is reasonable that the molecules responsible for the creation of a tumor-associated acidic microenvironments are regarded as targets for cancer therapy, because an acidic microenvironment is known to be involved in various biological behaviors of tumor cells, such as their proliferation [6], invasion and metastasis [7], [8], angiogenesis [9] and drug resistance. We investigated the effects of CMA, a V-ATPase inhibitor, on the cellular behavior of OSCCs in this study. We found that the CMA induced apoptosis in three of the four OSCC cell lines, while combination treatment was required to allow for the efficient induction of apoptosis in the fourth cell line. The induction of apoptosis by CMA involved the phosphorylation of p38, similar to the effects observed in human colon adenocarcinoma cells treated with bafilomycin A1, another V-ATPase inhibitor [40]. Although the acidic organelles in SQUU-B cells, one of the OSCCs used in this study, were disappeared after CMA treatment, the SQUU-B cells were resistant to CMA treatment. The phosphorylation of p38 in the CMA-treated SQUU-B cells was not altered, unlike the others, and the Bax/Bcl-2 ratio was dramatically lower in the CMA-treated SQUU-B cells compared to the other cells. The combined treatment with CMA and SAHA led to increase susceptibility of the SQUU-B cells to CMA-induced apoptosis at least partially via the downregulation of Bcl-2 expression.

In this study, the acidification of vesicular organelles in all four OSCC cell lines was prevented by the treatment with a low concentration of CMA. The CMA treatment decreased the cell viability and induced apoptosis in MISK81-5, SAS and HSC-4 cells, based on the results of MTS assay and the appearance of DNA fragmentation, as indicated by the DNA laddering and TUNEL staining. Yoshimoto et al. [45] reported that the induction of apoptosis in cancer cells by synthetic analogues of the concanamycins corresponds with the V-ATPase inhibitory activity of the analogues. An analogue without V-ATPase inhibitory activity did not induce apoptosis in the cancer cells. Therefore, CMA may inhibit the lysosomal functions by preventing the acidification of lysosomes in these CMA-sensitive OSCC cells, thus resulting in apoptosis in these cells, as was observed previously in colon cancer cells [40]. These findings also suggest that V-ATPase could be an attractive target for OSCCs. However, it seemed that the V-ATPase inhibition by CMA is not able to induce apoptosis in the CMA-resistant cells.

CMA activated p38, one of the MAPKs, in the CMA-sensitive cells. The activation of the p38 MAPK pathway and downregulation of a pro-survival protein (Bcl-2) significantly enhanced the anchorage-dependent tumor cell apoptosis in OSCC cells treated with tetrathiomolybdate [41]. Activation of the p38 MAPK pathway is suggested to be an excellent potential therapeutic strategy in esophageal squamous cell carcinoma [39] and other types of malignant tumors. Of a note, a p38 inhibitor partially attenuated the anti-proliferative effect of bafilomycin A1 on colon adenocarcinoma cells [40]. Our results also indicated that the phosphorylation of p38 is involved in the CMA-induced apoptosis in CMA-sensitive OSCC cells.

Interestingly, the SQUU-B cells showed chemoresistance to 10 nM CMA, although the 2.5 nM CMA treatment for 2 hr before acridine orange-staining caused the disappearance of the orange fluorescence in these cells, indicating that it still had potent effects on the intracellular pH. In the CMA-treated SQUU-B cells, the p-p38 expression levels was not altered, and the Bax/Bcl-2 ratio in the CMA-treated SQUU-B cells was dramatically lower than that in the CMA-sensitive OSCC cells. These results support the involvement of activated p38 in the induction of apoptosis by CMA, as mentioned above, and suggest that the upregulation of Bcl-2 expression by CMA plays an important role in the resistance to CMA-induced apoptosis in the SQUU-B cells.

This hypothesis is supported by a study of Rouette et al. [37] showing that the upregulation of Bcl-2 expression is associated with the resistance to cisplatin-induced apoptosis in endometrial cancer cells. The activation of PKC and Akt2 is involved in the pathway regulating Bcl-2. However, the detailed mechanism(s) by which the expression levels of proapoptotic and anti-apoptotic factors are regulated in the CMA-treated SQUU-B cells remain unknown. Because CMA did lead to limited anti-tumor effects on the SQUU-B cells, such as the disappearance of the orange fluorescence and a decrease in cell mobility (shown in Figs. 1 and S1, respectively), V-ATPase inhibition may not be the main cause of the apoptosis induced by CMA, or V-ATPase inhibition may not activate the apoptosis pathway in the CMA-resistant carcinoma cells. The apoptotic pathway induced by CMA may be different from mechanisms associated with the migration/invasion. Therefore, it will be necessary to clarify the mechanisms underlying the limitations of the efficacy of CMA in the SQUU-B cells in a future study. Consequently, the detection of putative regulatory factors would provide important insight into potential therapeutic strategies involving CMA (or V-ATPase in general).

In addition, few studies have so far addressed how apoptosis is induced in V-ATPase inhibitor-resistant tumor cells, although both CMA and bafilomycin A1 restore the sensitivity of the drug-resistant cells to anthracyclines such as daunomycin, doxorubicin and epirubicin [46]. Combination therapy using two or more chemotherapeutic agents is undertaken as a common strategy in current oncology to overcome the unsatisfactory efficacy associated with using a single drug. Therefore, SAHA, one of the HDACi, was used in the combination treatment with CMA in this study, because accumulated evidence has demonstrated that SAHA was capable of causing drug-induced apoptosis in OSCC cells, including carcinoma cells with resistance to antitumor drugs [42]–[44]. The combination therapy using CMA and SAHA was investigated in the CMA-resistant SQUU-B cells. SAHA significantly enhanced the cytotoxicity of CMA in the CMA-resistant SQUU-B cells based on the cell viability analysis. The SQUU-B cells treated with SAHA alone also exhibited a significant SAHA-dependent decrease in cell viability in comparison with that observed in the untreated SQUU-B cells. SAHA treatment alone also significantly reduced the expression of the Bax and Bcl-2 mRNAs in the SQUU-B cells. In the combination treatment, the expression level of Bax mRNA was gradually recovered, whereas the decrease of Bcl-2 expression was preserved. In the study by Dong et al. [47], 5 µM SAHA treatment also decreased the Bcl-2 expression in renal cells, and the decrease was observed to be even stronger following treatment with a higher concentration of SAHA, whereas the Bax and Bak expression levels remains constant.

To confirm whether Bcl-2 is a pivotal factor in the CMA-resistance in the SQUUB cells, we investigated the effect of a BH3 mimetic (HA14-1) [48], [49] on the SQUU-B cells treated with CMA. HA14-1 enhanced the cytotoxicity of CMA in the SQUU-B cells. Pretreatment with a siRNA targeting Bcl-2 also induced apoptosis in SQUU-B cells in response to CMA treatment. Based on these findings, it is conceivable that the combination treatment increased the Bax/Bcl-2 ratios and that this was responsible for induction of apoptosis in the CMA-resistant SQUU-B cells. These results support the idea that Bcl-2 plays a role in the resistance of SQUU-B cells to CMA. However, this study demonstrated that only one cell line (SQUU-B cells) was sensitized to CMA by the HDACi. In future studies, therefore, it will be necessary to use the other cells with resistance to CMA, and to clarify the detailed mechanisms underlying the increased susceptibility of the CMA-resistant cells to CMA-induced apoptosis induced by the combination of the HDAC inhibitor and CMA. In addition, the mechanism(s) responsible for the SAHA-induced Bcl-2/Bcl-XL in the study by Dong et al. [47] remained unclear.

Hence, we next examined the phosphorylation of STAT3, which is upstream of Bcl-2, and p38 in the SQUU-B cells treated with CMA and SAHA. The level of pY705-STAT3 was decreased by the treatment with SAHA alone, and by treatment with both CMA and SAHA, whereas the pS727-STAT3 and phosphorylation of p38 increased. In other studies, SAHA (vorinostat) reduced the pY705-STAT3 [50], [51], and the inhibition of pY705-STAT3 suppressed the Bcl-2 expression and enhanced the cytotoxicity of chemotherapeutic drugs to induce apoptosis in human cancer cell lines [52]. Andersson et al. [53] showed that insulin enhanced pS727-STAT3, but reduced pY705-STAT3 when it was used given together with IL-6. The pS727-STAT3 enhanced the dephosphorylation of pY705-STAT3, largely through the nuclear TC45 phosphatase [54]. Together, our results and those of previous studies suggest that pS727-STAT3 may negatively regulate pY705-STAT3 in the SQUU-B cells treated with SAHA. The negative interaction between the pS727 and pY705 of STAT3 may be involved in the reduction of the Bcl-2 expression. Additionally, the activation of the p38 MAPK pathway, followed by caspase-3 cleavage, is responsible for SAHA (vorinostat)-induced apoptosis in human breast cancer cells [55]. Activation of p38 also appears to participate in the activation of S727-STAT3 [56]. It is conceivable that the decrease of Bcl-2 expression induced by the change in the STAT3 phosphorylation can sensitize the cells to apoptotic injury. This phenomenon may be associated with not only transcriptional inhibition, but also translational inhibition, protein degradation, or both. However, Choi and Han [56] showed that the activation of STAT3 (pS727) increased the Bcl-2 expression in HeLa cells overexpressing phospholipase D, which was different from our results concerning the Bcl-2 expression following STAT3 activation. The role of pS727-STAT3 in regulation of STAT3 activity may therefore be dependent on the cell type, the specific activated kinase pathway and/or the cytokines/growth factors used [33], [53].

A gene expression analysis of ATP6V0A3, ATP6V0A4 and ATP6V1C1 of the V-ATPase subunits, as well as Bax and Bcl-2, was performed to make comparisons between the CMA-sensitive cell lines and high CMA-resistant SQUU-B cells. Interestingly, in the most CMA-sensitive MISK81-5 cells, the expression levels of the ATP6V0A3, ATP6V0A4 and ATP6V1C1 genes were significantly higher than those of the HaCaT cells. In the CMA-sensitive SAS and HSC-4 cells, the expression level of the ATP6V0A3 gene was significantly higher than that of the HaCaT cells. Meanwhile, in the CMA-resistant SQUU-B cells, the expression levels of the examined V-ATPase subunit genes were almost equal to or lower than those of the HaCaT cells. The Bax/Bcl-2 ratios in both the MISK81-5 and SQUU-B cells were markedly low. Thus, it seems that V-ATPase expression might be associated with the sensitivity/resistance to CMA in the carcinoma cells. However, because only one cell line with CMA resistance was used in this study, there is still no definitive answer at present with regard to what phenotype or molecular parameters can predict the level of sensitivity/resistance to CMA in carcinoma cells.

In conclusion, our present study provides evidence that a V-ATPase inhibitor, CMA, can induce apoptosis in human OSCC cells. Certain OSCC cell lines, such as SQUU-B cells may be resistant to CMA. In addition, combined treatment with CMA and SAHA led to a stronger anti-tumor effect in these CMA-resistant cells. A decrease in Bcl-2 expression resulting from the changes in STAT3 phosphorylation, such as a decrease of pY705 and an increase of pS727, is at least partially responsible for inducing the increased susceptibility of SQUU-B cells to CMA. Although the detailed mechanism(s) underlying the resistance to CMA in certain OSCC cells, and the induction of susceptibility to CMA by SAHA remain to be elucidated, the present findings provide important insight into the response of OSCC cells to CMA. Although *in vivo* studies, and eventually clinical studies, would be necessary to confirm our findings, the present results suggest that even in apparently CMA-resistant OSCC cells, a combination of CMA with SAHA may allow for efficient cancer therapy.

## Supporting Information

Figure S1 **Inhibition of the migration of CMA-resistant OSCC cells by CMA treatment. A & B.** SQUU-B cells with (**A**) or without (**B**) 20 nM CMA treatment were analyzed for cell migration using a wound-healing assay. The CMA treatment suppressed the migration of the SQUU-B cells in comparison to the untreated SQUU-B cells at 24 hr after the removal of the insert (Culture-Insert, Ibidi, WI, USA). **C**. The number of SQUU-B cells was counted within the cell-free space at 24 hr after the removal of the insert. The *in vitro* migration was decreased in the CMA-treated SQUU-B cells compared to control SQUU-B cells, suggesting that the CMA treatment is at least partly involved in the reduced invasiveness of the CMA-treated SQUU-B cells. The data are expressed as the percentage of the cell number normalized by that of the untreated SQUU-B cells (mean ± SD). **p<0.01 versus the untreated cells.
(TIF)

## References

[1] Licitra L, Grandi C, Guzzo M, Mariani L, Lo Vullo S, et al (2003) Primary chemotherapy in resectable oral cavity squamous cell cancer: a randomized controlled trial. J Clin Oncol 21: 327–333. 12525526 10.1200/JCO.2003.06.146

[2] Feng Z, Guo W, Zhang C, Xu Q, Zhang P, et al (2011) CCND1 as a predictive biomarker of neoadjuvant chemotherapy in patients with locally advanced head and neck squamous cell carcinoma. PLoS One 6: e26399 10.1371/journal.pone.0026399 22065993 PMC3204964

[3] Ma J, Liu Y, Yang X, Zhang CP, Zhang ZY, et al (2013) Induction chemotherapy in patients with resectable head and neck squamous cell carcinoma: a meta-analysis. World J Surg Oncol 11: 67 10.1186/1477-7819-11-67 23497185 PMC3601969

[4] Chidzonga MM (2006) Oral malignant neoplasia: a survey of 428 cases in two Zimbabwean hospitals. Oral Oncol 42: 177–183. 16256412 10.1016/j.oraloncology.2005.07.003

[5] Chidzonga MM, Mahomva L (2006) Squamous cell carcinoma of the oral cavity, maxillary antrum and lip in a Zimbabwean population: a descriptive epidemiological study. Oral Oncol 42: 184–189. 16256417 10.1016/j.oraloncology.2005.07.011

[6] Kraus M, Wolf B (1996) Implications of acidic tumor microenvironment for neoplastic growth and cancer treatment: a computer analysis. Tumour Biol 17: 133–154. 8638088 10.1159/000217977

[7] Kato Y, Nakayama Y, Umeda M, Miyazaki K (1992) Induction of 103-kDa gelatinase/type IV collagenase by acidic culture conditions in mouse metastatic melanoma cell lines. J Biol Chem 267: 11424–11430. 1317866

[8] Rofstad EK, Mathiesen B, Kindem K, Galappathi K (2006) Acidic extracellular pH promotes experimental metastasis of human melanoma cells in athymic nude mice. Cancer Res 66: 6699–6707. 16818644 10.1158/0008-5472.CAN-06-0983

[9] Koukourakis MI, Giatromanolaki A, Sivridis E, Bougioukas G, Didilis V, et al (2003) Lactate dehydrogenase-5 (LDH-5) overexpression in non-small-cell lung cancer tissues is linked to tumour hypoxia, angiogenic factor production and poor prognosis. Br J Cancer 89: 877–885. 12942121 10.1038/sj.bjc.6601205 PMC2394471

[10] De Milito A, Fais S (2005) Tumor acidity, chemoresistance and proton pump inhibitors. Future Oncol 1: 779–786. 16556057 10.2217/14796694.1.6.779

[11] Wahl M, Grant D (2000) Effects of microenvironmental extracellular pH and extracellular matrix proteins on angiostatin's activity and on intracellular pH. Gen Pharmacol 35: 277–285. 11888684 10.1016/s0306-3623(01)00115-x

[12] Ahmad KA, Iskandar KB, Hirpara JL, Clement MV, Pervaiz S (2004) Hydrogen peroxide-mediated cytosolic acidification is a signal for mitochondrial translocation of Bax during drug-induced apoptosis of tumor cells. Cancer Res 64: 7867–7878. 15520193 10.1158/0008-5472.CAN-04-0648

[13] Yang L, Mei Y, Xie Q, Han X, Zhang F, et al (2008) Acidification induces Bax translocation to the mitochondria and promotes ultraviolet light-induced apoptosis. Cell Mol Biol Lett 13: 119–129. 17965970 10.2478/s11658-007-0042-x PMC6275645

[14] Martínez-Zaguilán R, Raghunand N, Lynch RM, Bellamy W, Martinez GM, et al (1999) pH and drug resistance. I. Functional expression of plasmalemmal V-type H^+^-ATPase in drug-resistant human breast carcinoma cell lines. Biochem Pharmacol 57: 1037–1046. 10796074 10.1016/s0006-2952(99)00022-2

[15] Sennoune SR, Bakunts K, Martínez GM, Chua-Tuan JL, Kebir Y, et al (2004) Vacuolar H^+^-ATPase in human breast cancer cells with distinct metastatic potential: distribution and functional activity. Am J Physiol Cell Physiol 286: C1443–C1452. 14761893 10.1152/ajpcell.00407.2003

[16] Lu X, Qin W, Li J, Tan N, Pan D, et al (2005) The growth and metastasis of human hepatocellular carcinoma xenografts are inhibited by small interfering RNA targeting to the subunit ATP6L of proton pump. Cancer Res 65: 6843–6849. 16061667 10.1158/0008-5472.CAN-04-3822

[17] Chung C, Mader CC, Schmitz JC, Atladottir J, Fitchev P, et al (2011) The vacuolar-ATPase modulates matrix metalloproteinase isoforms in human pancreatic cancer. Lab Invest 91: 732–743. 21339745 10.1038/labinvest.2011.8 PMC3084324

[18] Nishisho T, Hata K, Nakanishi M, Morita Y, Sun-Wada GH, et al (2011) The a3 isoform vacuolar Type H^+^-ATPase promotes distant metastasis in the mouse B16 melanoma cells. Mol Cancer Res 9: 845–855. 21669964 10.1158/1541-7786.MCR-10-0449

[19] Sennoune SR, Luo D, Martínez-Zaguilán R (2004) Plasmalemmal vacuolar-type H+-ATPase in cancer biology. Cell Biochem Biophys 40: 185–206. 15054222 10.1385/CBB:40:2:185

[20] Ohta T, Numata M, Yagishita H, Futagami F, Tsukioka Y, et al (1996) Expression of 16 kDa proteolipid of vacuolar-type H(+)-ATPase in human pancreatic cancer. Br J Cancer 73: 1511–1517. 8664121 10.1038/bjc.1996.285 PMC2074554

[21] Ma L (1992) Center MS (1992) The gene encoding vacuolar H^+^-ATPase subunit C is overexpressed in multidrug-resistant HL60 cells. Biochem Biophys Res Commun 182: 675–681. 1370888 10.1016/0006-291x(92)91785-o

[22] Murakami T, Shibuya I, Ise T, Chen ZS, Akiyama S, et al (2001) Elevated expression of vacuolar proton pump genes and cellular PH in cisplatin resistance. Int J Cancer 93: 869–874. 11519050 10.1002/ijc.1418

[23] Chauhan SS, Liang XJ, Su AW, Pai-Panandiker A, Shen DW, et al (2003) Reduced endocytosis and altered lysosome function in cisplatin-resistant cell lines. Br J Cancer 88: 1327–1334. 12698203 10.1038/sj.bjc.6600861 PMC2747565

[24] Petrangolini G, Supino R, Pratesi G, Dal Bo L, Tortoreto M, et al (2006) Effect of a novel vacuolar-H+-ATPase inhibitor on cell and tumor response to camptothecins. J Pharmacol Exp Ther 318: 939–946. 16714402 10.1124/jpet.106.103481

[25] Shi Y, Tang B, Yu PW, Tang B, Hao YX, et al (2012) Autophagy protects against oxaliplatin-induced cell death via ER stress and ROS in Caco-2 cells. PLoS One 7: e51076 10.1371/journal.pone.0051076 23226467 PMC3511352

[26] Otero-Rey EM, Somoza-Martin M, Barros-Angueira F, García-García A (2008) Intracellular pH regulation in oral squamous cell carcinoma is mediated by increased V-ATPase activity via over-expression of the ATP6V1C1 gene. Oral Oncol 44: 193–199. 17467328 10.1016/j.oraloncology.2007.02.011

[27] García-García A, Pérez-Sayáns García M, Rodríguez MJ, Antúnez-López J, Barros-Angueira F, et al (2012) Immunohistochemical localization of C1 subunit of V-ATPase (ATPase C1) in oral squamous cell cancer and normal oral mucosa. Biotech Histochem 87: 133–139 10.3109/10520295.2011.574647 21526910

[28] Matsuo K, Ishibashi Y, Kobayashi I, Ozeki S, Ohishi M, et al (1994) New human oral squamous carcinoma cell line and its tumorigenic subline producing granulocyte colony-stimulating factor. Jpn J Cancer Res 85: 1257–1262. 7531680 10.1111/j.1349-7006.1994.tb02938.x PMC5919384

[29] Miyazaki K, Takaku H, Umeda M, Fujita T, Huang WD, et al (1990) Potent growth inhibition of human tumor cells in culture by arginine deiminase purified from a culture medium of a Mycoplasma-infected cell line. Cancer Res 50: 4522–4527. 2164440

[30] Takahashi K, Kanazawa H, Akiyama Y, Tasaki S, Takahara M, et al (1989) Establishment and characterization of a cell line (SAS) from poorly differentiated human squamous cell carcinoma of the tongue. J Jpn Stomatol Soc 38: 20–28.

[31] Morifuji M, Taniguchi S, Sakai H, Nakabeppu Y, Ohishi M (2000) Differential expression of cytokeratin after orthotopic implantation of newly established human tongue cancer cell lines of defined metastatic ability. Am J Pathol 156: 1317–1326. 10751357 10.1016/S0002-9440(10)65002-X PMC1876874

[32] Boukamp P, Petrussevska RT, Breitkreutz D, Hornung J, Markham A, et al (1988) Normal keratinization in a spontaneously immortalized aneuploid human keratinocyte cell line. J Cell Biol 106: 761–771. 2450098 10.1083/jcb.106.3.761 PMC2115116

[33] Naher L, Kiyoshima T, Kobayashi I, Wada H, Nagata K, et al (2012) STAT3 signal transduction through interleukin-22 in oral squamous cell carcinoma. Int J Oncol 41: 1577–1586 10.3892/ijo.2012.1594 22922995 PMC3583669

[34] Ookuma YF, Kiyoshima T, Kobayashi I, Nagata K, Wada H, et al (2013) Multiple functional involvement of thymosin beta-4 in tooth germ development. Histochem Cell Biol 139: 355–370 10.1007/s00418-012-1033-1 23052839

[35] Xiao YT, Xiang LX, Shao JZ. (2008) Vacuolar H(+)-ATPase. Int J Biochem Cell Biol 40: : 2002–2006. Review. 10.1016/j.biocel.2007.08.006 17897871

[36] Hinton A, Sennoune SR, Bond S, Fang M, Reuveni M, et al (2009) Function of a subunit isoforms of the V-ATPase in pH homeostasis and in vitro invasion of MDA-MB231 human breast cancer cells. J Biol Chem 284: 16400–16408 10.1074/jbc.M901201200 19366680 PMC2713521

[37] Rouette A, Parent S, Girouard J, Leblanc V, Asselin E (2012) Cisplatin increases B-cell-lymphoma-2 expression via activation of protein kinase C and Akt2 in endometrial cancer cells. Int J Cancer 130: 1755–1767. 21618512 10.1002/ijc.26183

[38] Aiko K, Tsujisawa T, Koseki T, Hashimoto S, Morimoto Y, et al (2002) Involvement of cytochrome c and caspases in apoptotic cell death of human submandibular gland ductal cells induced by concanamycin A. Cell Signal. 14: 717–722. 10.1016/s0898-6568(02)00016-5 12020772

[39] Lioni M, Noma K, Snyder A, Klein-Szanto A, Diehl JA, et al (2008) Bortezomib induces apoptosis in esophageal squamous cell carcinoma cells through activation of the p38 mitogen-activated protein kinase pathway. Mol Cancer Ther 7: 2866–2875 10.1158/1535-7163.MCT-08-0391 18790767 PMC2903039

[40] Wu YC, Wu WK, Li Y, Yu L, Li ZJ, et al (2009) Inhibition of macroautophagy by bafilomycin A1 lowers proliferation and induces apoptosis in colon cancer cells. Biochem Biophys Res Commun 382: 451–456 10.1016/j.bbrc.2009.03.051 19289106

[41] Kumar P, Yadav A, Patel SN, Islam M, Pan Q, et al (2010) Tetrathiomolybdate inhibits head and neck cancer metastasis by decreasing tumor cell motility, invasiveness and by promoting tumor cell anoikis. Mol Cancer 9: 206 10.1186/1476-4598-9-206 20682068 PMC2922193

[42] Rikiishi H, Shinohara F, Sato T, Sato Y, Suzuki M, et al (2007) Chemosensitization of oral squamous cell carcinoma cells to cisplatin by histone deacetylase inhibitor, suberoylanilide hydroxamic acid. Int J Oncol 30: 1181–1188. 17390020

[43] Suzuki M, Endo M, Shinohara F, Echigo S, Rikiishi H (2009) Enhancement of cisplatin cytotoxicity by SAHA involves endoplasmic reticulum stress-mediated apoptosis in oral squamous cell carcinoma cells. Cancer Chemother Pharmacol 64: 1115–1122 10.1007/s00280-009-0969-x 19280190

[44] Bruzzese F, Leone A, Rocco M, Carbone C, Piro G, et al (2011) HDAC inhibitor vorinostat enhances the antitumor effect of gefitinib in squamous cell carcinoma of head and neck by modulating ErbB receptor expression and reverting EMT. J Cell Physiol 226: 2378–2390 10.1002/jcp.22574 21660961

[45] Yoshimoto Y, Jyojima T, Arita T, Ueda M, Imoto M, et al (2002) Vacuolar-type H(+)-ATPase inhibitory activity of synthetic analogues of the concanamycins: is the hydrogen bond network involving the lactone carbonyl, the hemiacetal hydroxy group, and the C-19 hydroxy group essential for the biological activity of the concanamycins? Bioorg Med Chem Lett 12: 3525–3528. 12443768 10.1016/s0960-894x(02)00806-5

[46] Ouar Z, Bens M, Vignes C, Paulais M, Pringel C, et al (2003) Inhibitors of vacuolar H^+^-ATPase impair the preferential accumulation of daunomycin in lysosomes and reverse the resistance to anthracyclines in drug-resistant renal epithelial cells. Biochem J 370: 185–193. 12435274 10.1042/BJ20021411 PMC1223162

[47] Dong G, Wang L, Wang CY, Yang T, Kumar MV, et al (2008) Induction of apoptosis in renal tubular cells by histone deacetylase inhibitors, a family of anticancer agents. J Pharmacol Exp Ther 325: 978–984 10.1124/jpet.108.137398 18310471

[48] Manero F, Gautier F, Gallenne T, Cauquil N, Grée D, et al (2006) The small organic compound HA14-1 prevents Bcl-2 interaction with Bax to sensitize malignant glioma cells to induction of cell death. Cancer Res 66: 2757–2764. 16510597 10.1158/0008-5472.CAN-05-2097

[49] Nyhan MJ, O'Donovan TR, Elzinga B, Crowley LC, O'Sullivan GC, et al (2012) The BH3 mimetic HA14-1 enhances 5-fluorouracil-induced autophagy and type II cell death in oesophageal cancer cells. Br J Cancer 106: 711–718 10.1038/bjc.2011.604 22240779 PMC3322956

[50] Hashioka S, Klegeris A, McGeer PL (2012) The histone deacetylase inhibitor suberoylanilide hydroxamic acid attenuates human astrocyte neurotoxicity induced by interferon-γ. J Neuroinflammation 9: 113 10.1186/1742-2094-9-113 22647614 PMC3410763

[51] Karube K, Tsuzuki S, Yoshida N, Arita K, Kato H, et al (2013) Comprehensive gene expression profiles of NK cell neoplasms identify vorinostat as an effective drug candidate. Cancer Lett 333: 47–55 10.1016/j.canlet.2012.12.022 23348693

[52] Liu A, Liu Y, Jin Z, Hu Q, Lin L, et al (2012) XZH-5 inhibits STAT3 phosphorylation and enhances the cytotoxicity of chemotherapeutic drugs in human breast and pancreatic cancer cells. PLoS One 7: e46624 10.1371/journal.pone.0046624 23056374 PMC3463519

[53] Andersson CX, Sopasakis VR, Wallerstedt E, Smith U (2007) Insulin antagonizes interleukin-6 signaling and is anti-inflammatory in 3T3-L1 adipocytes. J Biol Chem 282: 9430–9435. 17267401 10.1074/jbc.M609980200

[54] Wakahara R, Kunimoto H, Tanino K, Kojima H, Inoue A, et al (2012) Phospho-Ser727 of STAT3 regulates STAT3 activity by enhancing dephosphorylation of phospho-Tyr705 largely through TC45. Genes Cells 17: 132–145 10.1111/j.1365-2443.2011.01575.x 22233524

[55] Uehara N, Kanematsu S, Miki H, Yoshizawa K, Tsubura A (2012) Requirement of p38 MAPK for a cell-death pathway triggered by vorinostat in MDA-MB-231 human breast cancer cells. Cancer Lett 315: 112–121 10.1016/j.canlet.2011.07.032 22093617

[56] Choi HJ, Han JS (2012) Overexpression of phospholipase D enhances Bcl-2 expression by activating STAT3 through independent activation of ERK and p38MAPK in HeLa cells. Biochim Biophys Acta 1823: 1082–1091 10.1016/j.bbamcr.2012.03.015 22504301

