# Supplementary figures and images for: Chemoresistance to Concanamycin A1 in Human Oral Squamous Cell Carcinoma Is Attenuated by an HDAC Inhibitor Partly via Suppression of Bcl-2 Expression
Source: PLoS One. 2013 Nov 20;8(11):e80998. doi: 10.1371/journal.pone.0080998 (PMC3835574; doi:10.1371/journal.pone.0080998)

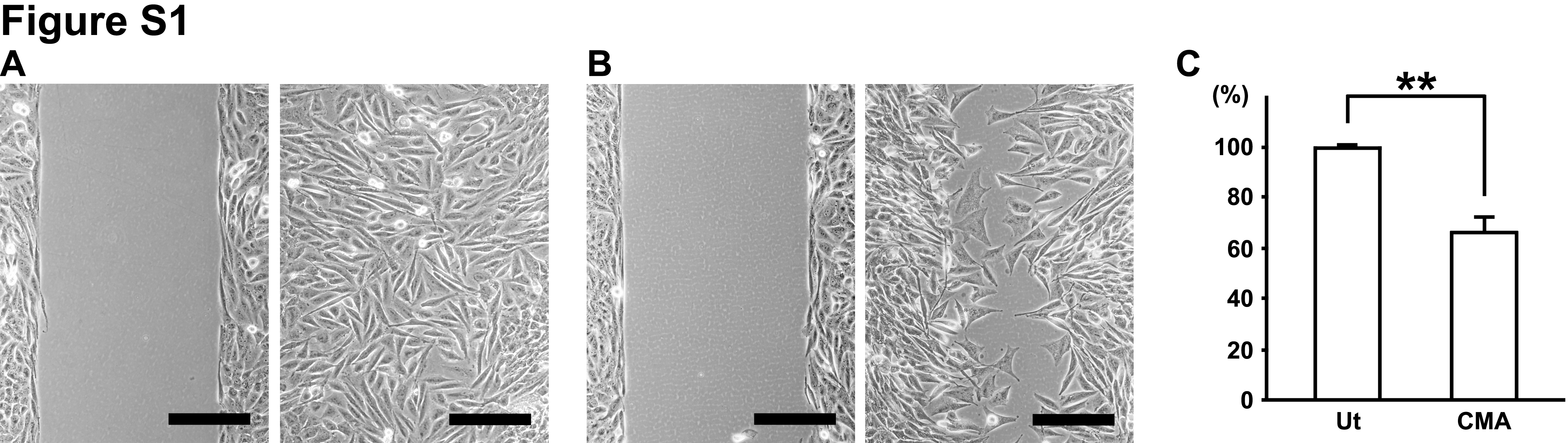

Supplement: Figure S1 — Inhibition of the migration of CMA-resistant OSCC cells by CMA treatment. A & B. SQUU-B cells with (A) or without (B) 20 nM CMA treatment were analyzed for cell migration using a wound-healing assay. The CMA treatment suppressed the migration of the SQUU-B cells in comparison to the untreated SQUU-B cells at 24 hr after the removal of the insert (Culture-Insert, Ibidi, WI, USA). C. The number of SQUU-B cells was counted within the cell-free space at 24 hr after the removal of the insert. The in vitro migration was decreased in the CMA-treated SQUU-B cells compared to control SQUU-B cells, suggesting that the CMA treatment is at least partly involved in the reduced invasiveness of the CMA-treated SQUU-B cells. The data are expressed as the percentage of the cell number normalized by that of the untreated SQUU-B cells (mean ± SD). **p<0.01 versus the untreated cells. (TIF) [file pone.0080998.s001.tif]
